# Regulation of emerging gene technologies in India

**DOI:** 10.1186/s12919-018-0106-0

**Published:** 2018-07-19

**Authors:** Vibha Ahuja

**Affiliations:** Biotech Consortium India Limited, New Delhi, India

## Abstract

In India, genetically modified organisms (GMOs) and the products thereof are regulated under the “Rules for the manufacture, use, import, export & storage of hazardous microorganisms, genetically engineered organisms or cells, 1989” (referred to as Rules, 1989) notified under the Environment (Protection) Act, 1986. These Rules are implemented by the Ministry of Environment, Forest and Climate Change, Department of Biotechnology and State Governments though six competent authorities. The Rules, 1989 are supported by series of guidelines on contained research, biologics, confined field trials, food safety assessment, environmental risk assessment etc.

The definition of genetic engineering in the Rules, 1989 implies that new genome engineering technologies including gene editing and gene drives. May be covered under the rules. India is a signatory to the Cartagena Protocol on Biosafety (CPB), however, the definition of modern biotechnology, as in CPB is yet to be adopted in the national regulations. The regulatory authorities review and take into account the experience by other countries in dealing with new technologies. However, there is yet no clarity on how the emerging technologies will be dealt with in India, though research has been initiated in several leading institutions.

## Background

India has a systematic and structured regulatory framework for biosafety evaluation of genetically modified organisms (GMOs) and products thereof. India was one of the early movers in development of a biosafety regulatory system for GMOs, way back in 1989. The apex rules for regulation of all activities related to GMOs are notified under Environment (Protection) Act, 1986. In addition, there are other acts, rules and policies which are also applicable to these organisms.

## Regulation of GMOs in India

An overview of biosafety regulations is presented below:

### Rules, 1989

In India, Ministry of Environment, Forest and Climate Change (MoEFCC) introduced the Environment (Protection) Act, 1986 as an umbrella legislation to provide a holistic framework for the protection and improvement to the environment. Thereafter, a series of Rules were notified to address various problems such as hazardous chemicals, hazardous wastes, solid wastes, biomedical wastes, etc.

In connection with the use of micro-organisms and application of gene technology, the MoEFCC notified the “Rules for manufacture, use/import/export & storage of hazardous microorganisms/genetically engineered organisms or cells, 1989” [1] as per powers conferred by Sections “Regulation of Genome Engineering Technologies in India”, 8 and 25 of Environment (Protection) Act, 1986. These rules are very broad in scope essentially covering entire spectrum of activities involving GMOs and products thereof. They also apply to any substances, products, and food stuffs, etc., of which such cells, organisms or tissues hereof form part. New gene technologies apart from genetic engineering have also been included. The gene technology and genetic engineering have been defined as follows in the text of the Rules, 1989.

(i) “Gene Technology” means the application of the gene technique called genetic engineering, include self-cloning and deletion as well as cell hybridization.
(ii) “Genetic engineering” means the technique by which heritable material, which does not usually occur or will not occur naturally in the organism or cell concerned, generated outside the organism, or the cell is inserted into said cell or organism. It shall also mean the formation of new combinations of genetic material by incorporation of a cell into a host cell, where they occur naturally (self-cloning) as well as modification of an organism or in a cell by deletion and removal of parts of the heritable material.

Rules, 1989 are implemented by MoEFCC jointly with the Department of Biotechnology (DBT), Ministry of Science & Technology and state governments. Six Competent Authorities and their composition have been notified under these Rules that includes:

1. rDNA Advisory Committee (RDAC)
2. Institutional Biosafety Committee (IBSC)
3. Review Committee on Genetic Manipulation (RCGM)
4. Genetic Engineering Appraisal Committee (GEAC)
5. State Biotechnology Coordination committee (SBCC)
6. District Level Committee (DLC)

While the RDAC is advisory in function, the IBSC, RCGM, and GEAC are responsible for regulating function. SBCC and DLC are for monitoring purposes.

The role of each of the committees is summarized in Table 1 and detailed below:

(i) **Recombinant DNA Advisory Committee (RDAC):** This committee constituted by the DBT takes note of developments in biotechnology at national and international levels. The RDAC is advisory in nature and expected to give recommendations from time to time on safety regulations in research and applications of GMOs and products thereof. This Committee prepared the Recombinant DNA Biosafety Guidelines in 1990, which were adopted by the Government for conducting research and handling of GMOs in India.
(ii) **Institutional Biosafety Committee (IBSC):** It is necessary that each institution intending to carry out research activities involving genetic manipulation of microorganisms, plants or animals should constitute the IBSC. The IBSCs comprise of the head of the organization, scientists engaged in DNA work, a medical expert and a nominee of the DBT. The IBSC is the nodal point for interaction within the institution for implementation of the guidelines.
(iii) **Review Committee on Genetic Manipulation (RCGM):** The RCGM functions as a body under the DBT to monitor the safety related aspects in respect of on-going research projects and activities involving GE organisms/hazardous microorganisms. RCGM is also mandated to bring out manuals of guidelines specifying procedure for regulatory process with respect to activities involving GE organisms in research, use and applications with a view to ensure environmental safety. The RCGM includes representatives of scientific departments/organizations in the country viz. Indian Council of Medical Research (ICMR), Indian Council of Agricultural Research (ICAR), Council of Scientific and Industrial Research (CSIR) and other experts in their individual capacity.
(iv) **Genetic Engineering Appraisal Committee (GEAC):** GEAC is the apex committee functioning in MoEFCC and has representatives from concerned ministries/agencies and experts. GEAC is chaired by a senior officer of MoEFCC and co-chaired by expert nominated by DBT. GEAC is responsible for approval of activities involving large scale use of hazardous microorganisms and recombinant products in research and industrial production from the environment angle.
(v) **State Biotechnology Coordination Committee (SBCC):** SBCC is constituted in each State where research and applications of GMOs are underway. SBCC is headed by the Chief Secretary of the State and has primarily monitoring responsibilities:
(vi) **District Level Committee (DLC):** DLCs are constituted in districts, wherever required to monitor the safety regulations in installation engaged in the use of GMOs/hazardous microorganisms and its applications in the environment. Each DLC is headed by the District Collector (officer responsible for administration of a district) with officers concerned with public health, environment, pollution control etc. at the district level.
  The interactive mechanisms between committees have also been provided in Rules, 1989. All IBSCs are required to review the applications and submit their recommendations and reports to RCGM. RCGM review and gives its recommendation for large scale activities, field trials and environmental release to GEAC. DLCs are also required to regularly submit its report to the SBCC/GEAC.

**Table 1:** Six competent authorities

Statutory committee	Function	Administrating agency
rDNA Advisory Committee (RDAC)	Advise on biosafety of emerging technologies	Department of Biotechnology, Ministry of Science and Technology
Institutional Biosafety Committee (IBSC)	R&D and Contained Experiments	Set up in registered Institutions, Universities and Private Companies; report to RCGM
Review Committee on Genetic Manipulation (RCGM)	Scientific risk assessment of plants, animals, biopharma, microbes and Guidelines	Department of Biotechnology, Ministry of Science and Technology
Genetic Engineering Appraisal Committee (GEAC)	Final Approval for environmental release including confined field trials	Ministry of Environment and Forests and climate change
State Biotechnology Coordination committee (SBCC)	For monitoring and supervision at state level	Concerned State Governments
District Level Committee (DLC)	Depending upon the need for local supervision and compliance	

**Fig. 1:**
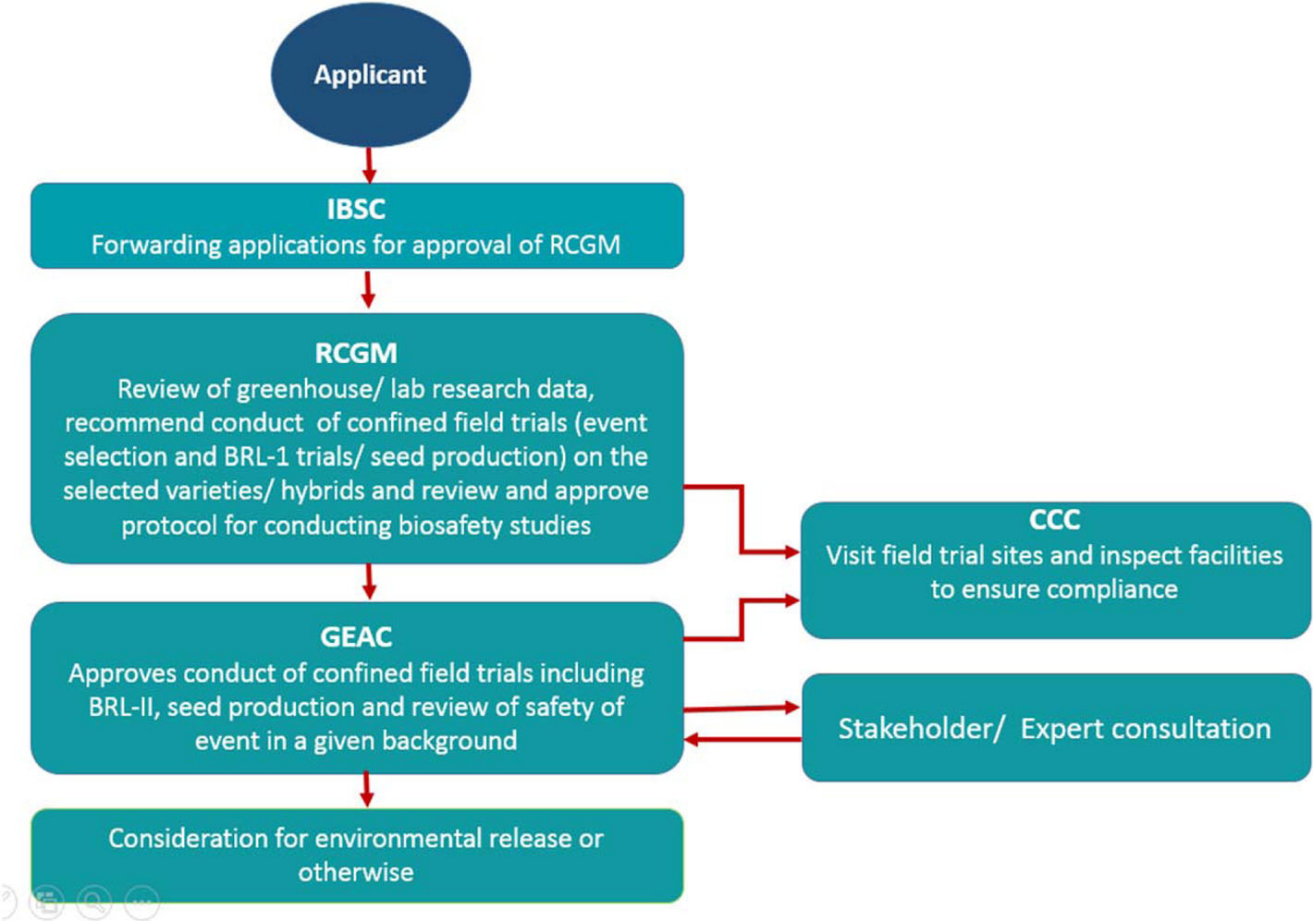
Procedure for approval of confined field trials and environmental release of GE plants

In addition to the above, various sub-committees and expert committees are constituted by RCGM and GEAC on a case by case basis. Such committees comprise of experts from various disciplines drawn from public sector institutions to prepare and review various guidelines and biosafety data. Central Compliance Committees are also set up for monitoring of confined field trials on case by case basis. An example of functioning of regulatory system for confined field trials and environmental release of a GE plant is illustrated in Fig. 1.

## Approvals and prohibitions

Rules, 1989 provide for compliance of the biosafety safeguards and any violation and non-compliance in this area attracts punitive actions provided under the EPA, 1986. The approvals and prohibitions under Rules 1989 are summarized below:

- No person shall import, export, transport, manufacture, process, use or sell any GMOs, substances or cells except with the approval of the GEAC.
- Use of pathogenic organisms or GMOs or cells for research purpose shall only be allowed in laboratories or inside laboratory areas notified for this purpose under the EPA, 1986.
- Any person operating or using GMOs for scale up or pilot operations shall have to obtain permission from GEAC.
- Experiments for the purpose of education involving GMOs can be undertaken with the oversight of IBSCs.
- Deliberate or unintentional release of GMOs not allowed.
- Production in which GMOs are generated or used shall not be commenced except with the approval of GEACAll approvals shall be for a period of 4 years at first instance renewable for 2 years at a time.
- GEAC shall have powers to revoke approvals in case of:
  i. Any new information on harmful effects of GMOs.
  ii. GMOs cause such damage to the environment as could not be envisaged when approval was given.
  iii. Non-compliance of any conditions stipulated by GEAC.

## Supervision and penalties

GEAC supervises the implementation of the terms and conditions laid down in connection with the approvals accorded by it. GEAC may carry out supervision through SBCC, DLC or any authorized person. If orders are not complied, SBCC/DLC may take suitable measures at the expenses of the person who is responsible. In case of immediate interventions to prevent any damage, SBCC and DLC can take suitable measures and the expenses incurred will be recovered from the person responsible.

Various notifications have also issued under Rules, 1989 from time to time to address issues such as empowering Seed Inspectors/Seed Analyst/Laboratories notified under Seed Act under Environment (Protection) Act, 1986, exempting certain categories of recombinant pharmaceutical products, GM food stuffs, ingredients in food stuffs, and additives from the purview of Rules, 1989.

### Other relevant rules and policies

In addition to Rules, 1989, other Acts and Rules, also refer to specific activities/products involving GMOs [2]. These include Plant Quarantine Order, 2003. Biological Diversity Act, 2002 and Food Safety and Standards Act, 2006. Table 2 summarizes relevant Acts/Rules regulating GMOs in India.

**Table 2:** Relevant acts and rules regulating GMOs in India

S. no.	Act/Rules	Implemented by	Activities covered
1.	Rules for the Manufacture, Use, Import, Export and Storage of Hazardous Micro-organisms/ Genetically Engineered Organisms or Cells, 1989 issued under EPA	Ministry of Environment, Forest and Climate Change	Cover entire spectrum of activities involving GMOs and products thereof including sale, storage, exportation, importation, production, manufacturing, packaging, etc. Food stuffs have been moved out of the purview of Rules, 1989 recently.
2.	Plant Quarantine (Regulation For Import Into India) Order 2003	Ministry of Agriculture & Farmers Welfare	Covers regulation of import of germplasm/ GMOs/transgenic plant material for research purpose.
3.	Biological Diversity Act, 2002	National Biodiversity Authority	Regulates the use of biological resources including genes used for improving crops and livestock through genetic intervention.
4.	The Food Safety and Standards Act, 2006	Food Safety and Standards Authority of India	Regulates manufacture, storage, distribution, sale and import of food which includes GM food.

### Guidelines for safety assessment of GMOs

A series of guidelines for safety assessment procedures to be followed at various stages of development of GMOs i.e. research, confined field trials, food safety assessment, and environmental risk assessment have been adopted under Rules, 1989 from time to time. Issuance of guidelines by regulatory authorities in India corresponds to the research and development activities. Recombinant DNA Safety Guidelines primarily focussing on research and development activities on GMOs, shipment and importation for laboratory research etc. were adopted in 1990 within a year of notification of Rules, 1989. These guidelines provide guidance on containment measures to be followed for various categories of organisms based on risk category. Appropriate practices, equipment and facilities necessary for safeguards in handling organisms, plants, and animals have been provided [3]. As the active research was initiated in transgenic plants in nineties in India, Guidelines for research in transgenic plants were issued in 1998 by DBT to provide guidance for recombinant DNA research on plants. The guidelines also provide for design of a greenhouse and safeguards to be followed in field trials [4]. Guidelines and Standard Operating Procedures for conduct of confined field trials of regulated GE Plants were issued by DBT and MoEFCC in 2008 [5].

Guidelines for the Safety Assessment of Foods Derived from Genetically Engineered (GE) Plants were prepared by the Indian Council of Medical Research and adopted by RCGM and GEAC in 2008 [6]. These guidelines provide guidance on principles and steps on food safety assessment of GE plants and are based on guidelines and principles of Codex Alimentarius Commission, 2003. A series of accompanying protocols were prepared by DBT [7]. The guidelines include three appendices on dossier preparation checklist, Codex Alimentarius principles for the risk analysis of foods derived from modern biotechnology, safety assessment of foods derived from GE plants modified for nutritional or health benefits and food safety assessment in situations of low level presence of GE plant material in food.

MoEFCC in association with the DBT has recently adopted a set of three documents to strengthen the environmental risk assessment of GE plants in India. These include Guidelines for the Environmental Risk Assessment of Genetically Engineered Plants, 2016; Environmental Risk Assessment of Genetically Engineered Plants: A Guide for Stakeholders and Risk Analysis Framework, 2016 [8]. The Risk Analysis Framework is based on the problem formulation approach. Concept and principles of Risk Assessment, Risk Management and Risk Communication have been explained in the Risk Analysis Framework. Guidelines for ERA of GE plants have been prepared in consonance with Annex III to the Cartagena Protocol on Biosafety, to which India is a signatory.

### Status of research and commercialization of GMOs in India

India has a very rich and innovative R&D pipeline, as evident from the fact that there are more than 500 active IBSCs in the country. Several public and private sector institutions are involved in the research and development of GE plants in India. In one of the surveys conducted by MoEFCC in 2014 under the Phase II Capacity Building Project on Biosafety, over 85 different plant species were identified as currently being used in experimental work, including plants used for food, livestock feed, fiber fuel and dietary or medicinal purposes [9]. A comprehensive list of all of the crop species identified by respondents in the survey is placed in Table 3.

**Table 3:** List of crop species in the R&D pipeline

*Amorphaphallus* Apple *Arabidopsis* Areca nut *Brassica caranata Brassica juncea Brassica napus Brassica nigra Brassica rapa* *Bacopa monnieri* Bamboo Banana Black gram Black pepper Brahmi *Brassica spp.* Brinjal Broccoli Cabbage Capsicum Cardamom Cassava Castor Casuarina Catharanthus Cauliflower	Cenchrus *Centaurea depressa* Chickpea Chili Cocoa Coconut Coffee Common bean Cotton Cowpea Cumin Dendrobian Dolichos *Eleusine coracana* Eucalyptus Field pea Finger millet Foxtail millet Garden pea Ginger *Gmelina* Green gram Ground nut Guava Isagbol Jatropha	*Lathyrus* Lucerne Maize Mango Medicinal plants *Melia* Millet Morus sp. Mung bean Mustard *Ocimum sanctum* Oilseed *brassicas* Okra Onion *Panicum* Papaya Pearl millet *Pennisetum* *Phalaris minor Phillanthus Picorhhiza kurroa* Pigeon pea Pomegranate Poplar Potato Red pepper	Rice Rubber tree) Safflower Scented rose Sesame Sorghum Soybean Spinach Stevia Sugarcane Sunflower Sweet potato *Swertia spp.* *Switennia mehagony* Tea Teak Tobacco Tomato Triticale Turmeric Vegetables Watermelon Wheat *Withania somnifera*

Out of the above, more than 20 plants with varying traits such as hybrid seed production, insect resistance, herbicide tolerance, abiotic stress tolerance, nutritional enhancement, etc. are under various stages of field trials [10]. A list of GE plants for which confined field trials have been approved in the past 4 years is placed below Table 4.

**Table 4:** An indicative list of GE plants approved for confined field trials in India in last 4 years

S. N.	Plant	Trait	Organization
2.	Brinjal	Insect resistance	- Indian Agricultural Research Institute - Maharashtra Hybrid Seeds Company Limited (MAHYCO) - Bejo Sheetal Seeds
3.	Cabbage	Insect resistance	- Sungro Seeds
4.	Castor	Insect resistance	- Indian Institute of Oilseeds Research
5.	Cauliflower	Insect resistance	- Sungro Seeds
6.	Chickpea	Abiotic stress tolerance, insect resistance	- Indian Institute of Pulses Research - International Crops Research Institute for the Semi-Arid Tropics (ICRISAT) - MAHYCO
7.	Corn	Insect resistance, herbicide tolerance	- Monsanto - Pioneer - Dow Agrosciences
8.	Cotton	Insect resistance, herbicide tolerance	- Central Institute of Cotton Research - MAHYCO - Dow Agrosciences
9.	Groundnut	Virus resistance, abiotic stress tolerance	- ICRISAT
10.	Mustard	Hybrid seed production	- University of Delhi South Campus
11.	Okra	Insect resistance	- MAHYCO
12.	Papaya	Virus resistance	- Indian Institute of Horticultural Research
13.	Pigeon pea	Insect resistance	- Indian Institute of Pulses Research - MAHYCO
14.	Potato	Tuber sweetening, fungal resistance	- Central Potato Research Institute
15.	Rice	Insect resistance, diseases resistance, hybrid seed production, nutritional enhancement	- Indian Agricultural Research Institute - Tamil Nadu Agricultural University - Bayer Bioscience - MAHYCO - BASF India Ltd.
16.	Rubber	Abiotic stress tolerance	- Rubber Research Institute of India
17.	Sorghum	Insect resistance, abiotic stress tolerance	- Indian Institute of Millets Research
18.	Sugarcane	Insect resistance	- Sugarcane Breeding Institute
19.	Tomato	Insect resistance, virus resistance, fruit ripening	- Indian Institute of Vegetable Research - National Research Centre on Plant Biotechnology - MAHYCO
20.	Watermelon	Virus resistance	- Indian Institute of Horticultural Research
21.	Wheat	Effect of mutant strains Azotobacter	- National Research Centre on Plant Biotechnology

To date, Bt cotton is the only GE plant approved for commercial cultivation in India.

In addition to the above, research involving GM microorganisms for healthcare and industrial application under contained conditions is underway in several organizations. Indian companies are actively involved in products of similar biologics (also referred as biosimilars).

Regarding research in GE insects, limited efforts have been made by an Indian company for taking forward from technology from male sterile mosquitoes developed by Oxitec. The company has completed mating compatibility experiments with Indian strains and set up facilities for contained trials.

Development of GE silkworms is being actively pursued and field trials have been undertaken. All these applications are being dealt with on a case by case basis and no separate guidelines have been issued so far by the regulators.

### Regulation of genome engineering Technologies in India

Recognising the global advances in the area of genome engineering technologies and huge potential for practical applications in healthcare and agriculture, initiatives have been taken in India by leading research institutions. In 2014, DBT has constituted a dedicated Task Force on “Genome Engineering Technologies and their Applications” with a vision to foster innovation and promote development of Genome-wide Analysis and Engineering Technologies to make them accessible and affordable for wider use in life sciences. It is proposed to strengthen facilities on emerging technologies such as gene editing and support research projects. Efforts are also underway to develop human resource to catch up with the growth in this area and harness the benefits of these technologies for basic or applied use in larger context and drive research for technology development towards basic and applied use.

Regarding regulation of genome engineering technologies, there is still a debate in the country. The definition of GE technology as in Rules, 1989 is very broad based and include “modification, deletion or removal of parts of heritable material”. This implies that all new technologies will be subject to regulation under provision of Rules, 1989.

## Conclusions

As India is a signatory to Cartagena Protocol on Biosafety, the definition of modern biotechnology as in the Protocol was proposed to be adopted in the Bill for setting up of Biotechnology Regulatory Authority of India in 2013. However, the bill was lapsed and further action on the same is awaited.

In view of the present definition, it is expected that regulatory considerations for new and emerging technologies will be on a case by case basis based on the existing regulatory framework i.e. Rules, 1989 in the near future.
